# ﻿Acanthocephalans from freshwater fishes in northeast Thailand with the description of a new species of the subgenus *Acanthosentis* Verma & Dutta, 1929 (Acanthocephala, Quadrigyridae)

**DOI:** 10.3897/zookeys.1233.136533

**Published:** 2025-04-03

**Authors:** Olga Lisitsyna, Mikuláš Oros, Alexis Ribas, Srisupaph Poonlaphdecha, Daniel Barčák

**Affiliations:** 1 Department of Parasitology, Schmalhausen Institute of Zoology, Ukrainian National Academy of Sciences, Kiev, Ukraine Schmalhausen Institute of Zoology, Ukrainian National Academy of Sciences, Kiev Kiev Ukraine; 2 Institute of Parasitology, Slovak Academy of Sciences, Košice, Slovakia Institute of Parasitology, Slovak Academy of Sciences Košice Slovakia; 3 Parasitology Section, Department of Biology, Healthcare and Environment, Faculty of Pharmacy and Food Science, University of Barcelona, Barcelona, Spain University of Barcelona Barcelona Spain; 4 Institut de Recerca de la Biodiversitat (IRBio), Universitat de Barcelona, Barcelona, Spain Universitat de Barcelona Barcelona Spain

**Keywords:** Acanthogyrus (Acanthosentis), *Arhythmorhynchus*, DNA, fish helminths, integrative taxonomy, *Pallisentis*, *Raosentis*

## Abstract

During an ichthyoparasitological survey in northeast Thailand in 2015, four species of acanthocephalans were found in four species of freshwater fishes. Adult stages of *Pallisentisrexus* Wongkham & Whitfield, 1999 and *Raosentis* sp. (Eoacanthocephala, Quadrigyridae) were collected from *Channastriata* (Anabantiformes, Channidae) and *Mystusmysticetus* (Siluriformes, Bagridae), respectively, and cystacanths of *Arhythmorhynchus* sp. (Palaeacanthocephala, Polymorphidae) were found on the visceral organs of *M.albolineatus* (Siluriformes, Bagridae). Adult acanthocephalans of the subgenus Acanthosentis Verma & Dutta, 1929 isolated from *Barbonymusschwanenfeldii* (Cypriniformes, Cyprinidae) were morphologically distinct from all described species of the subgenus in the arrangement of rings of tegumental spines in two fields with a more or less pronounced distance between them, and by the presence of a dome-shaped process with a ring of small spines at the base at the posterior end in females. Molecular data were generated for three species and phylogenetic analysis based on the 18S rDNA clearly supported the generic identification of Acanthogyrus (Acanthosentis) barbonymi**sp. nov.** and *P.rexus.* While the phylogenetic position of the former species within the genus could not be clarified, the latter species formed a well-supported sister lineage in a clade with isolates of four congeneric species. Acanthogyrus (Acanthosentis) barbonymi**sp. nov.** is formally described, the first genetic data for *P.rexus* are generated, a species of the genus *Raosentis* Datta, 1947 is reported for the first time outside the Indian subcontinent, and a key to the species of the latter genus is presented.

## ﻿Introduction

Besides their classic recognition as causative agents of human and animal diseases, parasites are also integral components of ecosystems where they play remarkable roles in food webs, affect behavior, fitness, and survival of their hosts, and thus significantly contribute to forming a community structure (Hudson et al. 2006). Considering helminths, acanthocephalans are among the most neglected group despite their global distribution and often high abundances, complex life cycles which cross-link different trophic levels, and other interesting attributes that make them promising models in studies of evolution of parasitism, host-parasite interactions, and ecotoxicology (Sures et al. 1999; Near 2002; Perrot-Minnot et al. 2023). Some species may also be pathogenic to their hosts, including humans (Mathison et al. 2021).

Acanthocephalans have been reported in several studies on fish parasites in Thailand, most of them dealing with commercial fish species (Wongkham and Whitfield 1999, 2004; Mard-Arhin et al. 2001; Lerssutthichawal and Supamattaya 2005; Yooyen et al. 2006; Chaiyapo et al. 2007; Purivirojkul and Areechon 2008; Sriwongpuk 2017; Juntaban et al. 2021), rarely with non-commercial ones (Tunya and Wongsawad 2019). In total, 826 species of freshwater and brackish fish belonging to 88 families have been recorded in Thailand (Froese and Pauly 2024) and acanthocephalans have been reported from only 18 freshwater fish species, belonging to ten families. In many of these faunistic papers, the identification was not supported with descriptions or drawings, nor with molecular data. Several studies have focused on the distribution or the ecology of acanthocephalans (Pearse 1933; Farooqi and Sirikanchana 1987; Wongkham and Whitfield 1999, 2004; Amin and Taraschewski 2003; Wahab et al. 2021).

To date, four species of acanthocephalans have been found in freshwater fishes in Thailand: *Pallisentisnagpurensis* (Bhalerao, 1931) from the Asian swamp eel *Monopterusalbus* (Zuiew) and Bronze featherback *Notopterusnotopterus* (Pallas); *P.ophiocephali* (Thapar, 1930) from Striped snakehead *Channastriata* (Bloch); *P.rexus* Wongkham & Whitfield, 1999 from *Ch.striata* and *M.albus*; and Acanthogyrus (Acanthosentis) siamensis (Farooqi & Sirikanchana, 1987) from the Silver barb *Barbonymusgonionotus* (Bleeker) (= *Puntiusgonionotus*). Additionally, six acanthocephalans have been identified to genera: Acanthogyrus (Acanthosentis) sp., *Arhythmorhynchus* sp., *Pallisentis* sp, *Polyacanthorhynchus* sp., *Polymorphus* sp., and *Sphaerechinorhynchus* sp. (Mard-Arhin et al. 2001; Yooyen et al. 2006; Purivirojkul and Areechon 2008; Phalee 2018; Juntaban et al. 2021).

This paper presents data on an ichthyoparasitological survey in northeast Thailand in 2015, which were generated by an integrative approach combining alpha taxonomy and molecular phylogeny. The original findings involve the description of one new species of the subgenus Acanthosentis, the first record of the genus *Raosentis* outside India, and the first genetic data of the widespread species *Pallisentisrexus.* A key for identification for *Raosentis* species and phylogenetic analysis of the family Quadrigyridae are also presented.

## ﻿Materials and methods

### ﻿Specimen collection and morphological examination

The specimens studied in the present work were collected in Udon Thani and Nong Khai provinces of Thailand in April and May 2015 by examination of freshly captured fishes. Acanthocephalans were washed in tap water, kept in the refrigerator overnight to erect the proboscis, and fixed in non-denatured 70% ethanol.

For light microscopy, temporary slides mounted in Berlese’s medium were prepared. Line drawings were made using a drawing tube of Leica DM 5000B light microscope (Leica Microsystems, Wetzlar, Germany). All measurements in the text are in micrometers (μm) unless otherwise stated. Trunk length does not include proboscis, neck, or evaginated bursa. The width of the body is given as the maximum width. The ordinal number of the hooks in the longitudinal row is indicated in brackets when describing the dimensions of the blades and the roots of the hooks.

For scanning electron microscopy, specimens were dehydrated in an ethanol series and dried in hexamethyldisilazane (HMDS). Subsequently, the specimens were sputter coated with gold and captured with a JEOL JSM 6510LA (JEOL Ltd., Tokyo, Japan).

The scientific and common names of the fish hosts follow FishBase (Froese and Pauly 2024). Selected specimens have been deposited in the Helminthological collections of the Institute of Parasitology, Biology center of the Czech Academy of Sciences, Budweiss, Czechia (**IPCAS**) and the Natural History Museum, London, UK (**NHMUK**) (see Suppl. material 1).

### ﻿Molecular phylogenetic analysis

Total genomic DNA was extracted with Qiagen DNeasy Blood & Tissue kit from the middle part or posterior half of the body of the four *Acanthosentis* and three *Pallisentis* specimens (i.e., hologenophores); a complete cystacanth of *Arhythmorhynchus* was used as paragenophore (see Pleijel et al. 2008). The PCR amplification was targeted on three ribosomal nuclear markers and mitochondrial cytochrome c oxidase I (COI) using the primers of Garey et al. (1996) for 18S rRNA gene, ZX1 and 1500R primers (Olson et al. 2003; Bray et al. 2009) for 28S rRNA gene, LCO 1490 and HCO 02198 (Folmer et al. 1994) for partial COI gene. The primers used for amplification of the complete ITS region, forward (5´-GGAAGTAAAAGTCGTAACAAG-3´) and reverse (5´-GATATGCTTAARTTCAGCGGG-3´), are reversed/complementary versions of ZX1 and WormB (Littlewood and Olson 2001; Bray et al. 2009). The PCR products were verified on agarose gel by electrophoresis and enzymatically purified (Werle et al. 1994). The templates were sequenced by the Sanger method, at least two raw reads were *de-novo* aligned to create contiguous sequences, which were manually inspected for ambiguous positions. Newly generated sequences were deposited in the GenBank database (https://www.ncbi.nlm.nih.gov/genbank/).

The phylogenetic relationships within the family Quadrigyridae (Gyracanthocephala) were calculated based on 18S rRNA gene, the marker with reasonable number of available sequences. The dataset was aligned using MAFFT v. 7.490 employing the algorithm E-INS-i (Katoh et al. 2002; Katoh and Standley 2013). The 18S alignment, after manual removal of misaligned regions due to gaps, was 1,375 bp long. The optimal nucleotide substitution model TN+F+G4 was calculated in ModelFinder (Chernomor et al. 2016; Kalyaanamoorthy et al. 2017) using AICc criterion. Phylogeny was estimated in IQtree 2.0.5. using ultrafast bootstrapping of 1,000 replicates (Hoang et al. 2018; Minh et al. 2020).

## ﻿Results

### ﻿Species descriptions

**Eoacanthocephala**

**Quadrigyridae**

#### ﻿ Acanthogyrus (Acanthosentis) barbonymi sp. nov.

Taxon classificationAnimaliaGyracanthocephalaQuadrigyridae

6EE6D155-1C52-5FF6-9CC3-DD57B31A515C

https://zoobank.org/C3E9FFBA-329F-4D0B-B70E-FDDB85AC2909

Figs 1 , 2 , 3

##### Type host.

Tinfoil barb *Barbonymusschwanenfeldii* (Bleeker) (Cypriniformes, Cyprinidae).

##### Type locality.

Nong Khai Inland Fisheries Research and Development Center, Had Sai Thong village, Nong Khai province, Thailand (17°55.318'N, 102°36.230'E).

##### Site of infection.

Intestine.

##### Infection rates.

Prevalence 87.0%, intensity 7–67.

##### Type material.

Deposited in the Helminthological collections of IPCAS and NHMUK (Coll. nos. IPCAS A-145 and NHMUK 2025.1.8.1-15).

##### Molecular data.

The sequences of nuclear 18S rRNA (1,767 bp), 28S rRNA (1,188 bp) and the mitochondrial COI (657 bp) genes of Acanthogyrus (Acanthosentis) barbonymi sp. nov. were deposited in the GenBank database (Acc. nos. PQ636375–PQ636378, PQ636383–PQ636385, PQ631040, PQ631041).

##### Etymology.

Species name is derived from the scientific name of the host.

##### Morphology.

**Figure 1.**
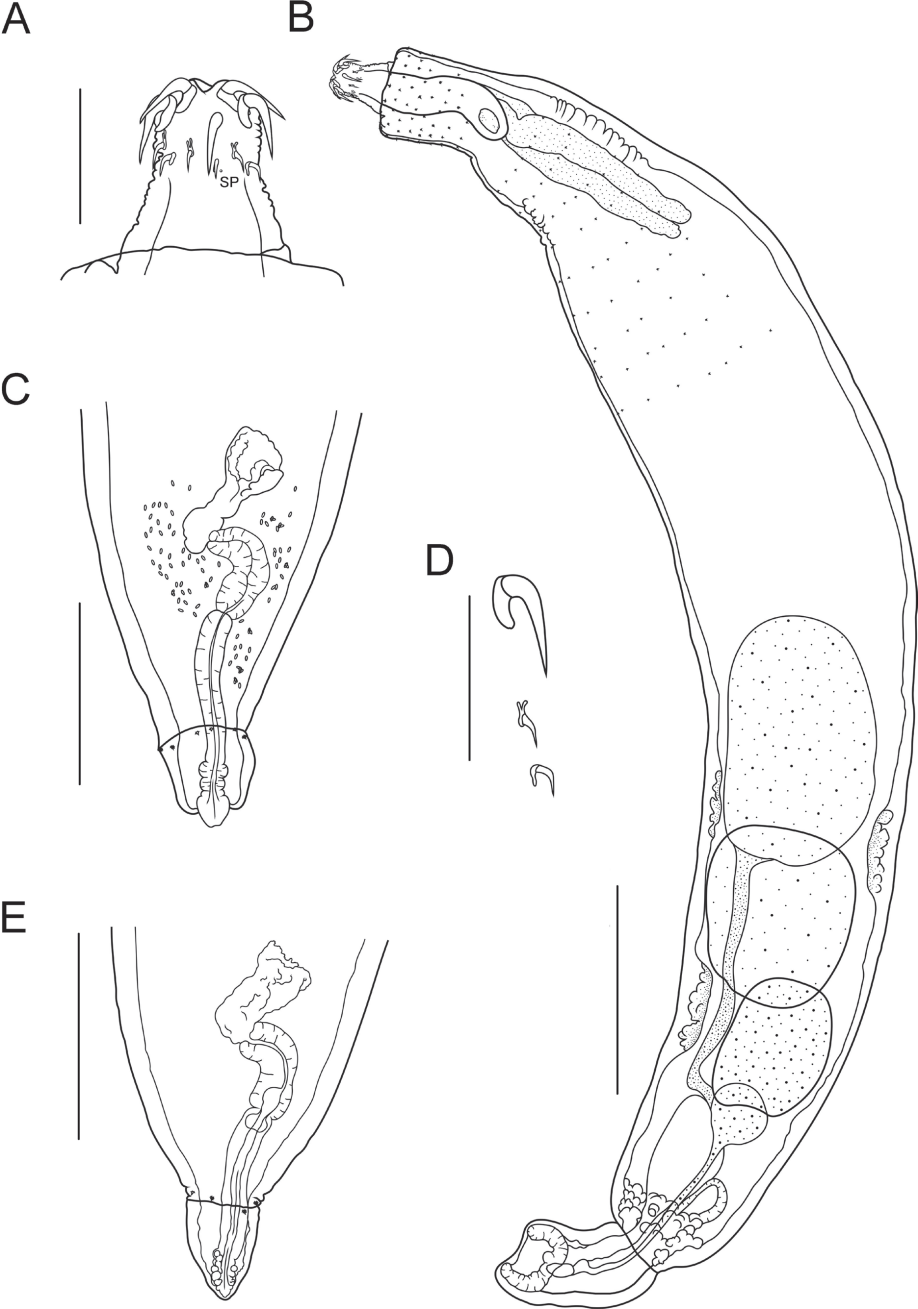
Line drawing of Acanthogyrus (Acanthosentis) barbonymi sp. nov. ex *Barbonymusschwanenfeldii* (Bleeker) from Thailand **A** proboscis of male, note sensory pore **B** total view of male **C** posterior end of mature female **D** hooks of proboscis **E** posterior end of immature female. Abbreviations: SP – sensory pore. Scale bars: 100 µm (**A, D**); 500 µm (**B, C, E**).

**Figure 2.**
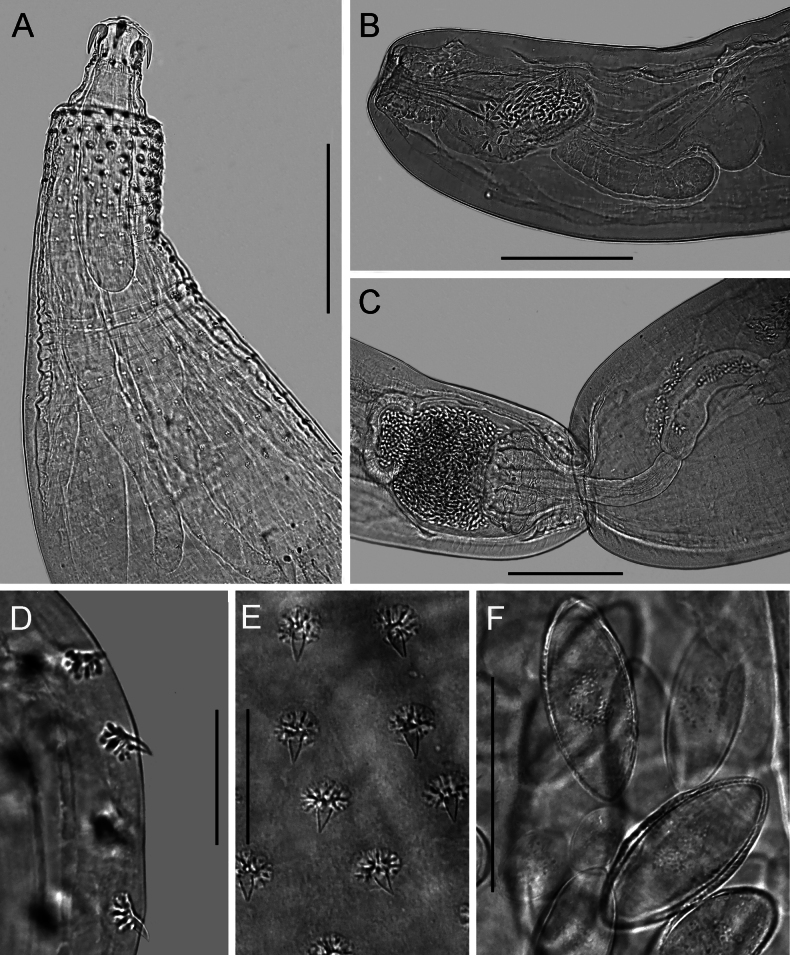
Light microscope photographs of Acanthogyrus (Acanthosentis) barbonymi sp. nov. ex *Barbonymusschwanenfeldii* (Bleeker) from Thailand **A** anterior part of female **B** posterior part of male with eggs in the cavity of the bursa **C** posterior ends of both sexes during copulation **D, E** tegumental spines **F** eggs. Scale bars: 300 µm (**A–C**); 50 µm (**D–F**).

**Figure 3.**
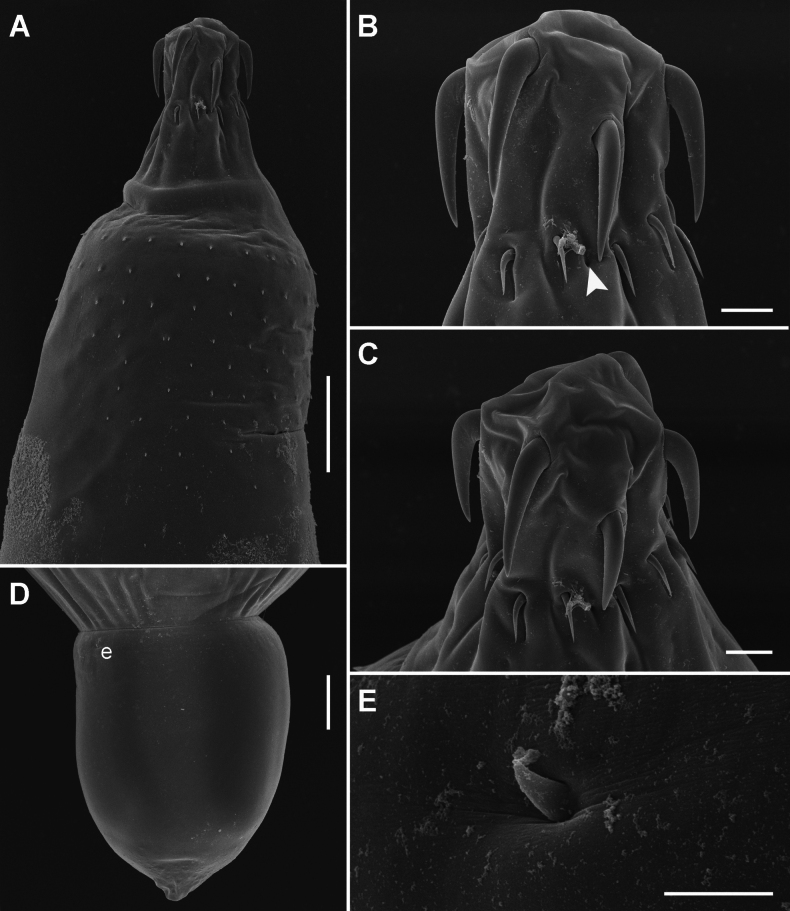
Scanning electron micrographs of Acanthogyrus (Acanthosentis) barbonymi sp. nov. ex *Barbonymusschwanenfeldii* (Bleeker) from Thailand **A** anterior part of female **B** lateral view on proboscis of female, note the sensory pore (arrowhead) **C** subapical view on proboscis of female **D** caudal process of female posterior end **E** tiny spine at base of caudal process. Scale bars: 100 µm (**A**); 20 µm (**B, C**); 50 µm (**D**); 5 µm (**E**).

Quadrigyridae with features of genus *Acanthogyrus* and subgenus Acanthosentis. Acanthocephalans white in color, medium size, usually sickle-shaped, with maximum width in anterior third of body (Figs 1–3). Female larger than male. Anterior body part with two fields of tegumental spines in rings with rosette-shaped root processes (Figs 1B, 2A, D, E). Number of giant tegumental nuclei not constant. Proboscis small, round, armed with 18 hooks in three rows, six hooks in each row. Sensory pore in base of proboscis (Figs 1A, B, 3B). Hooks of the anterior row large, with simple massive roots directed posteriorly, located irregularly, three slightly anteriorly, three slightly posteriorly (Fig. 1D). Hooks of middle row twice as small as hooks of anterior row, with complex forked roots directed anteriorly. Hooks of basal row smallest, with simple roots directed posteriorly (Fig. 1A, D). Neck conical. Proboscis receptacle with single-layer muscular wall, with cephalic ganglion at bottom. Lemnisci almost equal in length, extending to middle of body. Genital pore terminal in both sexes (Figs 1, 2B, C, 3D).

**Male** (based on nine mature specimens with sperm, measurements of the holotype specimens followed with measurements of type series). Trunk 3.54 (2.78–4.93) mm long, 618 (585–921) wide (Fig. 1B). Tegumental spines of anterior field with nine (9–12) rings, 16 (16–20) spines in each ring. Posterior rings of anterior field incomplete dorsally, basal ring with 2–10 spines. Length of spine blades 13 (12–16), diameter of rosette root 15 (12–16). Distance between spines fields (154–189). Somatic spines of posterior field with nine (8–10) rings, 12 (12–15) spines in each ring. Posterior rings of posterior spines field incomplete dorsally, basal ring with five or six spines. Length of spine blades 7 (7–14), diameter of rosette root 14 (12–24). Body wall with six (5–7) giant tegumental nuclei, four (2–4) dorsal, and two (1–3) ventral. Proboscis 73 (73–99) × 108 (108–138). Length of hook blades of anterior row 62–63 (51–69), middle 26 (21–26), basal 18 (18–24). Length of hook roots of anterior row 26–28 (25–45), middle 19 (16–20), basal 10 (10–15). Neck 74 (72–110) long, wide in anterior part 86 (86–105), in posterior part 126 (126–163). Proboscis receptacle 424 (370–467) × 91 (91–155). Lemnisci do not reach anterior testis, 874 (483–1,499) × 71 (44–92) and 834 (473–1,561) × 76 (44–98). Organs of reproductive system in posterior half of body. Testes oval, in tandem, anterior larger than posterior. Anterior testis 620 (429–1,078) × 399 (313–605), posterior testis 477 (378–702) × 356 (221–590). Cement gland oval, adjacent to posterior edge of posterior testis, 339 (267–575) × 302 (261–628). Almost round cement reservoir posteriorly to cement gland, 171 (121–280) × 179 (153–312). Saefftigen’s pouch club-shaped, 249 (432–621) × 82 (82–173). Vas deferens elongated, 393 (393–474) × 124 (119–124). Type specimen with leaf-shaped penis 94 × 42. Evaginated bursa 361 (335–361) × 229 (229–343).

**Female** (based on 13 specimens, 7 with eggs, 6 without eggs). Trunk 5.60–11.72 mm long, 0.86–1.45 mm wide. At base of caudal process 4–8 very small spines in one ring (Fig. 3E). Tegumental spines of anterior field with 10 or 11 rings, 16–22 spines in each ring. Posterior rings of anterior field incomplete dorsally, basal ring with 8–11 spines. Length of spine blades 12–15, diameter of rosette root 15–20. Distance between spines fields 100–262. Somatic spines of posterior field with 10–12 rings, 13–17 spines in each ring. Number of spines in ring decreases towards basal ring to 3–12. Length of spine blades 10–13, diameter of rosette root 13–20. Body wall with 6–10 giant tegumental nuclei, 2–6 dorsal, 2–5 ventral. Proboscis 112–136 × 133–170 (Figs 1A, 3C). Length of hook blades of anterior row 59–78, middle 28–34, basal 26–27. Length of hook roots of anterior row 33–45, middle 18–31, basal 15–17. Neck 42–129 long, width of anterior part 105–124, width of posterior part 166–320. Proboscis receptacle 361–560 × 124–147. Lemnisci 1.29–1.54 mm long, 83–154 wide. Female reproductive tract in posterior part of body, 962–1,400 long. Vagina with two sphincters (Fig. 1C, E). Eggs fusiform, elongate, no polar prolongation of fertilization membrane, 21–24 × 8–10 (Fig. 2F). Posterior end of female forms somewhat pronounced dome-shaped process, 180–343 × 198–356 (Fig. 3D) with complete or incomplete ring of 4–8 small spines at its base. At the moment of copulation, the male’s bursa embraces the dome-shaped caudal process of the female (Fig. 2C), and the eggs are injected into the cavity of the male bursa. After copulation, the bursa invaginates and some of the eggs may temporarily remain in cavity of bursa (Fig. 2B).

##### Remarks.

To date, 57 species have been described in the subgenus Acanthosentis of the genus *Acanthogyrus*, mainly parasites of freshwater fish in South and Southeast Asia (Amin 2005, 2013; Naidu 2012; Saxena et al. 2013; Amin et al. 2017; Mohd-Agos et al. 2021; Rana and Kaur 2023). Acanthogyrus (Acanthosentis) barbonymi sp. nov. differs from most species of the subgenus in the arrangement of rings of tegumental spines in two fields with a more or less pronounced distance between them, as well as the presence of a dome-shaped process with a ring of tiny spines at the base at the posterior end of the females. The arrangement of spines in two fields is characteristic for two species of the subgenus Acanthosentis, A. (A.) multispinus (Wang, 1966), described from the silver carp *Hypophthalmichthysmolitrix* (Valenciennes) from China (Wang 1966) and A. (A.) bispinosa Rana & Kaur, 2023 from the mrigal carp *Cirrhinusmrigala* Hamilton and the orangefin labeo *Labeocalbasu* Hamilton in Malaysia. However, A. (A.) barbonymi sp. nov. differs from A. (A.) multispinus in two characteristics: i) posterior rings of tegumental spines of A. (A.) barbonymi sp. nov. are incomplete in both fields, and the rings of the posterior field do not extend to the middle of the body; while the rings of spines in both fields of A. (A.) multispinus are complete and the rings of the posterior field of spines reach the posterior end of the body; ii) the proboscis hooks of the middle row A. (A.) barbonymi sp. nov. are half the size of the hooks of the apical row; while in A. (A.) multispinus, the size of the proboscis hooks gradually decreases from the apical to the basal row. A. (A.) barbonymi sp. nov. differs from A. (A.) bispinosa in the number of rings of spines, with 9–12 rings of spines in the anterior field, 8–11 rings of spines in the posterior field versus 7–10 rings of anterior spines and 23–38 rings of posterior spines in A. (A.) bispinosa (Rana and Kaur 2023).

One species of the subgenus Acanthosentis, A. (A.) siamensis (Farooqi & Sirikanchana, 1987) Amin, 2005, has been found in the silver barb *Barbonymusgonionotus* (Bleeker) (= *Puntiusgonionotus*) in Thailand (Farooqi and Sirikanchana 1987). Acanthogyrus (A.) barbonymi sp. nov. and A. (A.) siamensis have similarities in the size of the proboscis hooks and the shape of the female caudal process; however, they differ in the number of rings of body spines: 20–26 in A. (A.) barbonymi sp. nov. vs 3–4 in A. (A.) siamensis.

Recently, Mohd-Agos et al. (2021) described three new species of the subgenus Acanthosentis from *Barbonymusschwanenfeldii* from Lake Kenyir in Malaysia, namely A. (A.) kenyirensis Mohd-Agos, Mohd-Husin, Zakariah, Yusoff, Wahab, Jones, Hassan, 2021, A. (A.) tembatensis Mohd-Agos, Mohd-Husin, Zakariah, Yusoff, Wahab, Jones, Hassan, 2021 and *A.* (*A.*) *terengganuensis* Mohd-Agos, Mohd-Husin, Zakariah, Yusoff, Wahab, Jones, Hassan, 2021. Although described from the same fish host, A. (A.) barbonymi sp. nov. differs from these three species in the size of the proboscis hooks, and the hooks of the middle and basal rows of A. (A.) barbonymi sp. nov. have similar lengths and are approximately half as long as the hooks of the anterior row, whereas in A. (A.) kenyirensis, A. (A.) tembatensis, and A. (A.) terengganuensis the hooks of the anterior and middle rows are of comparable length and more than twice as long as the hooks of the basal row. Additionally, A. (A.) barbonymi sp. nov. clearly differs from them in other morphological characters: i) A. (A.) barbonymi sp. nov. has 4–11 giant nuclei in the tegument whereas A. (A.) kenyirensis and A. (A.) terengganuensis have no giant nuclei in the tegument; ii) the tegumental spines of A. (A.) barbonymi sp. nov. form two fields, with 9–12 rings of spines in the anterior field and 8–11 rings of spines in the posterior field whereas tegumental spines in A. (A.) kenyirensis and A. (A.) tembatensis are in one field with eight or nine rings; iii) A. (A.) kenyirensis, A. (A.) tembatensis, and A. (A.) terengganuensis have a unique collar ring on the neck and a muscular-like structure on both sides of proboscis attached to the ring whereas this structure absent in A. (A.) barbonymi sp. nov.; iv) females of A. (A.) barbonymi sp. nov. have a caudal process with tiny spines in one ring at its base whereas details of the caudal process in females of the three species from Malaysia were not mentioned. These morphological differences suggest that A. (A.) barbonymi sp. nov. is not conspecific with A. (A.) kenyirensis, A. (A.) tembatensis, nor A. (A.) terengganuensis.

Mohd-Agos et al. (2021) generated a sequence of ITS region from each of the three Malayan species (MK184204, MK184205, MK069588; 589–813 bp); a single COI sequence of A. (A.) kenyirensis (MN833316; 633 bp) was submitted to the GenBank by these authors, but this was not included in their work. COI sequences of our hologenophore specimens were almost identical (99.0% pairwise similarity) with the unpublished sequence of A. (A.) kenyirensis. The ITS marker could not be used for reliable phylogenetic analysis because we were unable to generate sequences of sufficient length. However, comparison of short (131 bp long) ITS sequences of our three hologenophores showed the highest pairwise similarity of A. (A.) barbonymi sp. nov. with A. (A.) terengganuensis (95.8%), followed by A. (A.) kenyirensis (89.2%) and A. (A.) tembatensis (83.2%). Since the analyses of ITS and COI markers provided inconsistent results, this could indicate misidentification of the specimens from Malaysia used for genotyping.

#### ﻿ Pallisentis rexus

Taxon classificationAnimaliaGyracanthocephalaQuadrigyridae

Wongkham & Whitfield, 1999

36639A52-0992-5E77-8B45-9CFAB236FCE1

Figs 4 , 5

##### Host.

Striped snakehead *Channastriata* (Bloch) (Anabantiformes, Channidae).

##### Locality.

Nong Samrong Lake, Nong Samrong Town, Udon Thani province, Thailand (17°27.065'N, 102°45.791'E) and a fish farm in Kong Nang village, Tha Bo Town, Nong Khai province, Thailand (17°54.190'N, 102°35.211'E).

##### Site of infection.

Intestine.

##### Infection rates.

Prevalence 84.9%, intensity 1–35.

##### Molecular data.

The nuclear18S rRNA (1,735 bp), 28S rRNA (1,085 bp), ITS (774 bp) and the mitochondrial COI (623 bp) genes sequences of *Pallisentisrexus* were deposited in the GenBank database (Acc. nos. PQ636379–PQ636381, PQ636386–PQ636388, PQ636390, PQ636391, PQ631042–PQ631044).

##### Morphology

**Figure 4.**
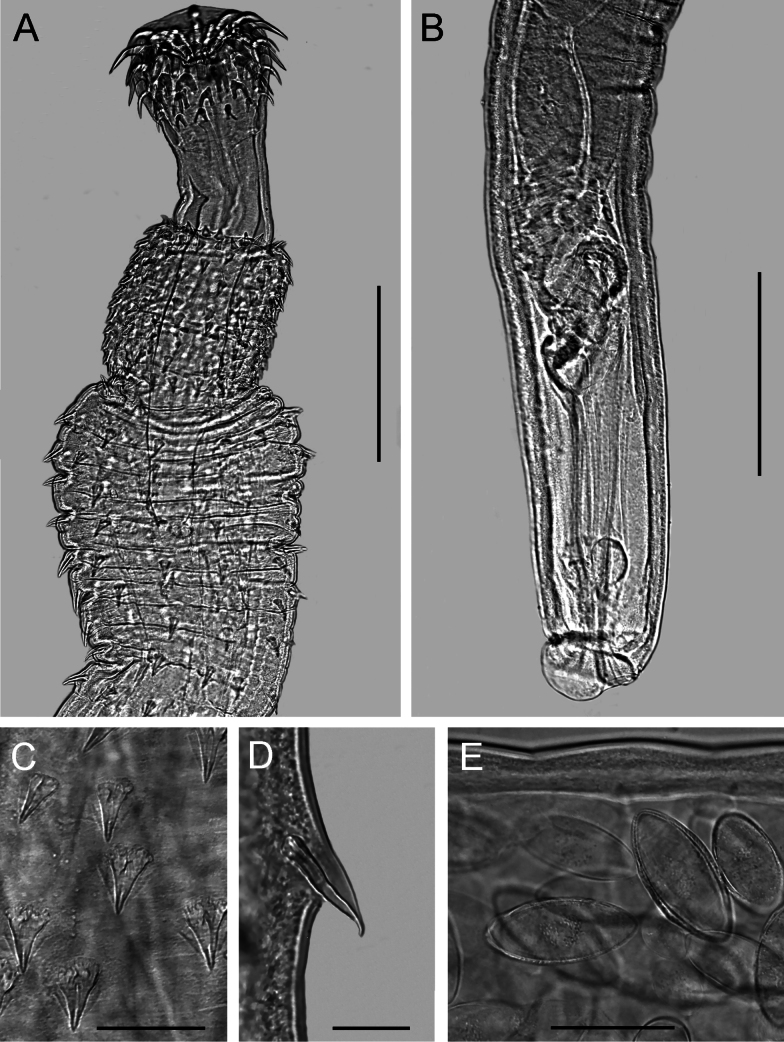
Light microscope photographs of *Pallisentisrexus* Wongkham & Whitfield, 1999 ex *Channastriata* (Bloch) from Thailand **A** anterior part of female **B** posterior part of male **C** tegumental spines of anterior field **D** tegumental spines of posterior field **E** eggs. Scale bars: 300 µm (**A, B**); 50 µm (**C–E**).

**Figure 5.**
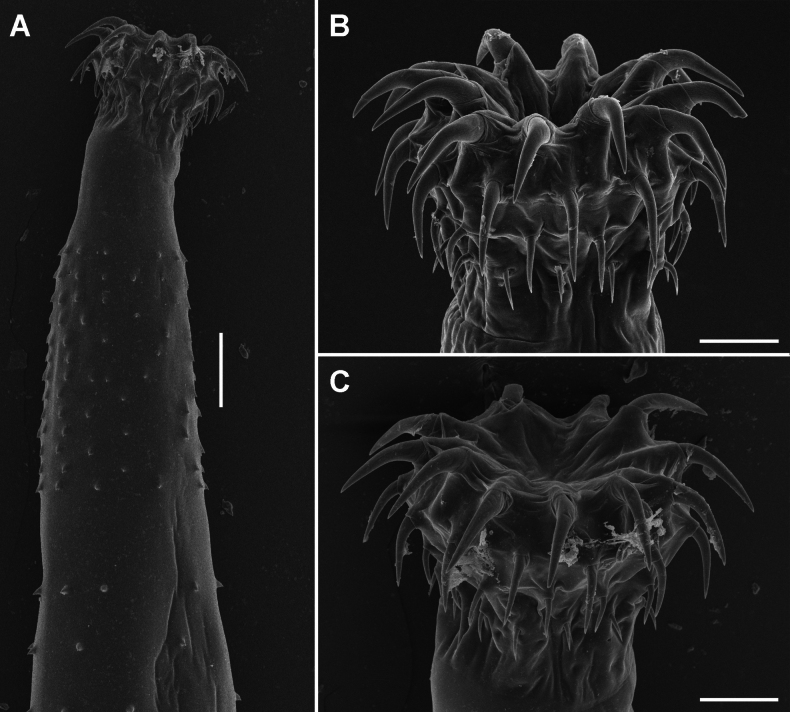
Scanning electron micrographs of *Pallisentisrexus* Wongkham & Whitfield, 1999 ex *Channastriata* (Bloch) from Thailand **A** anterior part of male **B, C** two views on proboscis of male. Scale bars: 100 µm (**A**); 50 µm (**B, C**).

(based on 5 males, 7 females). Medium-sized, white acanthocephalans. Anterior part of trunk with two fields of spines in rings (Figs 4A, 5A). Distance between anterior and posterior fields 105–151. Proboscis length less than its width (Fig. 4A). Proboscis with four rows of hooks, 12 hooks in each row. Hooks size decreases from apical to basal row. Proboscis receptacle with single-layer muscular wall, with cephalic ganglion in middle part. Neck conical. Lemnisci longer than proboscis receptacle. Gonopore terminal in both sexes.

**Male.** Trunk 4.03–7.3 mm long, 389–446 wide. Tegumental spines of anterior field with 14 rings, 14–18 spines in each. Length of spines 27–31 (Fig. 4C). Somatic spines of posterior field in 20–32 rings, anterior rings complete, ten spines in each, posterior rings incomplete, 2–4 in each. Length of spines 27–38 (Fig. 4D). Proboscis 164–214 × 253–289 (Figs 4A, 5B). Length of hook blades of anterior row 69–81, second 59–69, third 38–42, basal 25–33. Length of hook roots of anterior row 47–58, second 39–44, third 30–49, basal 19–25. Proboscis receptacle 419–594 × 98–160. Neck 164–186 long. Lemnisci 921–1,359 × 70–98. Organs of reproductive systems in posterior half of trunk (Fig. 4B). Testes I 575–695 × 172–200, II 636–654 × 125–198. Single cement gland 1,079 × 115–183, cement reservoir 121–432 × 153–200. Saefftigen’s pouch club-shaped, 307–478 × 115–125.

**Female.** Trunk 10.00–12.55 mm long, 264–405 wide. Tegumental spines of anterior field with 12–15 rings, anterior rings complete, 14–20 spines in each, posterior rings incomplete dorsally, 6–14 in each. Length of spines 25–34. Somatic spines of posterior field with 40–47 rings, ten spines in anterior rings, 3–11 in posterior rings. Length of spines 39–48. Proboscis 110–216 × 206–319. Length of hook blades of anterior row 74–85, second 63–74, third 45–52, basal 31–39. Length of hook roots of anterior row 48–67, second 43–65, third 35–46, basal 27–29. Proboscis receptacle 520–760 × 105–166. Neck 184–275 long. Lemnisci 1,094–1,115 × 55–72. Reproductive tract 398–531. Egg fusiform, elongate, no polar prolongation of fertilization membrane, 92–102 × 42–53 (Fig. 4E).

##### Remarks.

*Pallisentisrexus* (Eoacanthocephala, Quadrigyridae) was described from the striped snakehead *Channastriata* (Bloch) in the Chiang Mai Basin in Thailand (Wongkham; Whitfield, 1999). Later, immature specimens of this species were found in the Asian swamp eel, *Monopterusalbus* (Zieuw) from Bangkok, Thailand (Amin; Taraschewski 2003). Adult *P.rexus* were also found in *Channa* sр. from a river in northern Taiwan (Lisitsyna et al. 2023).

#### ﻿ Raosentis

Taxon classificationAnimaliaGyracanthocephalaQuadrigyridae

sp.

F5E8B903-AA0C-509F-A7C6-19D2F7E38461

Figs 6 , 7A

##### Host.

*Mystusmysticetus* Roberts (Siluriformes, Bagridae).

##### Locality.

Flood area of the Dan canal near Daeng Ban Non Du village, Udon Thani Province, Thailand (17°32.891'N, 103°03.831'E).

##### Site of infection.

Intestine.

##### Infection rates.

Prevalence 12.5% (1/8), intensity 1.

##### Morphology

(based on one male with sperm). Quadrigyridae with characters of genus *Raosentis*: i) proboscis with four rows of hooks with an unequal number of hooks in anterior and posterior rows; ii) a large area without hooks between second and third rows of proboscis hooks.

**Figure 6.**
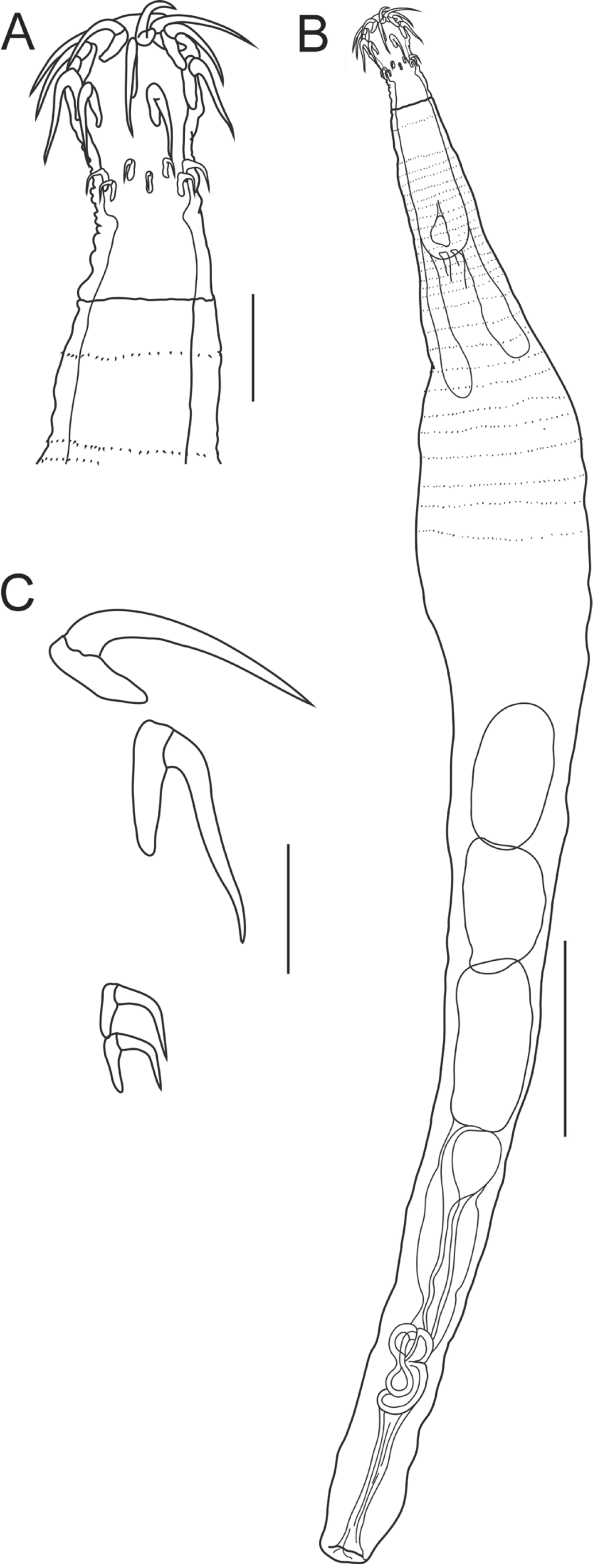
Line drawing of *Raosentis* sp. ex *Mystusmysticetus* Roberts from Thailand **A** anterior part of male **B** total view of male **C** hooks of proboscis. Scale bars: 100 µm (**A**); 500 µm (**B**); 50 µm (**C**).

**Male.** Small acanthocephalan, white, fusiform, 4.11 mm long, 471 maximum width in anterior quarter of body (Fig. 6B). Anterior part of body with in 28 rings of small tegumental spines, 50–54 spines in each ring. Field of spines 1.16 mm long beyond level of posterior edges of lemniscus, do not reach level of anterior margin of anterior testis. Distance between anterior ring of spines and next rings 77. Distance between 2^nd^ and 17^th^ rings of spines 18–22, distance between rings of spines increases posteriorly to 55–61. Proboscis 186 × 206, with 27 hooks in 4 rows, 6 or 7 spines in each row (Fig. 6A, C). Hooks of anterior row located irregularly, three slightly anteriorly, three slightly posteriorly. Hooks of two anterior rows large, with simple roots directed posteriorly (Fig. 4A, C). Their blades and roots 2–3 × larger than blades and roots of two posterior rows. Hooks of third and basal rows separated from hooks of the two anterior rows by 35, their roots also simple and directed posteriorly. Hooks blades of anterior row 91, second 78–92, third 20–29, basal 17–21. Hook roots of anterior row 46, second 50–52, third 21–23, basal 17–20. The neck is pronounced, 74 long, width in anterior part 87, in posterior part 135. Proboscis receptacle 524 × 129, with single-layer muscular wall, with cephalic ganglion 79 × 47 at bottom. Lemnisci 796 × 67, extend beyond proboscis receptacle, not reaching level of anterior edge of anterior testis. Organs of reproductive system 2.45 mm long, occupying 60% of body length, in its posterior part. Testes oval, tandem, anterior larger than posterior. Anterior testis 423 × 216, posterior 401 × 208. Cement gland elongated, 443 × 167, adjacent to posterior edge of posterior testis. Pear-shaped cement reservoir under cement gland, 190 × 72, branching posteгiorly into two vas deferens. Saefftigen’s pouch absent. Bursa in invaginated state 685 long. Gonopore terminal.

##### Remarks.

To date, all seven species of the genus *Raosentis* Datta, 1947 have been described from freshwater fishes in India (Naidu 2012). *Raosentis* sp. from Thailand differs from all other species of the genus by a slender elongated body more than 4 mm in length in the male (the body length of the male of other species of the genus does not exceed 3.3 mm) (Naidu 2012) as well as by the significant distance of the anterior rings of tegumental spines from the following rings of spines. *Raosentis* sp. morphologically resembles *R.lucknowensis* Saxena, Gupta, Johri, Ramakant, 2014, described from *Mystusvittatus* (Bloch) in River Gomti (Lucknow, India) by the number of rings of tegumental spines (28 in *Raosentis* sp., 28–30 in *R.lucknowensis*, in other species of the genus the number does not exceed 20) and by the length of the blades of the proboscis hooks of the two anterior rows (78–92 in our specimen, 70–90 in *R.lucknowensis*) (Saxena et al. 2014). However, these two species differ in the number of proboscis hooks (6, 7, 7, 7 in *Raosentis* sp. vs 8, 8, 8, 8 in *R.lucknowensis*), the size of the blades of the third and fourth rows (20–29 and 17–21 in *Raosentis* sp. vs 30–40 and 30–40 in *R.lucknowensis*, respectively). We suppose that *Raosentis* sp. might be a new species of the genus *Raosentis*, however, we waive its formal description since only a single specimen was studied. At the same time, we consider it appropriate to present a key for identifying the species of the genus.

### ﻿Key to species of the genus *Raosentis*

**Table:** 

1	Anterior ring of tegumental spines at a clear distance from the next rings of spines. Male body length is > 4 mm	***Raosentis* sp.**
–	Spines located in the anterior body in a continuous field. Body length of male ≤ 3.5 mm	**2**
2	Spines in anterior body in 9–12 rings	**3**
–	Spines in anterior body in 16–30 rings	**4**
3	Spines in anterior body in 9 rings. Organs of male reproductive system occupy 2/3 of body length, anterior testis at a distance from bottom of the proboscis sheath and posterior edge of the lemniscus	***R.thapari* Rai, 1967**
–	Spines in anterior body in 12 rings. Organs of male reproductive system occupy 3/4 of body length, anterior edge of anterior testis at level of posterior end of proboscis receptacle and posterior edge of the lemnisci	***R.godavarensis* Vankara & Vijayalakshmi, 2009**
4	Lemnisci unequal in length, extending to posterior end of proboscis receptacle	***R.cavasii* Gupta 2021**
–	Lemnisci equal in length, extending beyond posterior end of proboscis receptacle	**5**
5	The field of spines extends to level of anterior testis	**6**
–	The field of spines does not reach level of anterior testis	**7**
6	Tegument has 4 or 5 giant nuclei dorsally and 2 or 3 ventrally. Length of blades of anterior hooks 60–65 μm	***R.dattai* Gupta & Fatma, 1986**
–	Tegument has 3 giant nuclei dorsally and 3 ventrally. Length of blades of anterior hooks 80–90 μm	***R.lucknowensis* Saxena, Gupta, Johri & Ramakant, 2013**
7	Proboscis with 8–10 hooks in a row. Length of hook blades 60–90 μm, 50–58 μm, 30–46 μm, 24–35 μm respectively	***R.ivaniosi* George & Nadakal, 1978**
–	Proboscis with 6 or 7 in a row. Length of hook blades 85–115 μm, 70–95 μm, 25–35 μm, 25–30 μm respectively	***R.podderi* Datta, 1947**

#### ﻿Palaeacanthocephala

**Polymorphidae**

##### ﻿ Arhythmorhynchus

Taxon classificationAnimaliaGyracanthocephalaQuadrigyridae

sp.

91BA2147-DA66-596B-A9E8-1DEB0914EDFE

Fig. 7B

###### Host.

*Mystusalbolineatus* Roberts (Siluriformes, Bagridae).

###### Locality.

Flood area of the Dan canal near Daeng Ban Non Du village, Udon Thani Province, Thailand (17°32.891'N, 103°03.831'E).

###### Site of infection.

Body cavity.

###### Infection rates.

Prevalence 1/1, intensity 6.

###### Molecular data.

Nuclear 18S rRNA (1,703 bp), 28S rRNA (1,132 bp), ITS (847 bp), and mitochondrial COI (603 bp) genes sequences of *Arhythmorhynchus* sp. were deposited in the GenBank database (Acc. nos. PQ636382, PQ636389, PQ636392, PQ631045).

###### Note.

Six cysthacanths of the genus *Arhythmorhynchus* were found in the body cavity of one bagrid catfish *Mystusalbolineatus* Roberts. Five of them were in capsules with invaginated proboscis, one cysthacanth, female, had an evaginated proboscis, facilitating its morphological examination.

###### Morphology.

Trunk 1.27 mm long with maximum width at level of middle of proboscis receptacle 391. The front part of body with one field of spines. Its extent same ventrally and dorsally. Spines blades 21 long. Cylindrical proboscis with expansion in middle part. Proboscis 513 × 157 with 16 longitudinal rows of hooks, 22–23 hooks in each row. Sizes of hooks do not differ dorsally and ventrally. Hooks in anterior eight or nine rows large, with simple massive roots directed posteriorly. Hooks in next 13 rows spine-shaped, with short roots processes directed posteriorly. Length of hook blades: 33–40 (hook 2), 35–41 (3), 37–40 (4–7), 38–41 (8), 32–34 (9), 20–22 (10), 17–21 (11), 15–19 (12), 16–20 (13), 16–19 (14), 16–18 (15), 16–17 (16), 16 (17–23). Length of hook roots: 23–29 (2), 31 (3), 32–33 (4), 32 (5), 34–39 (6), 39–41 (7), 40–43 (8), 27–39 (9). Length of root processes of next hooks 8–12. Hooks in last one or two rows without roots. Neck retracted. Proboscis receptacle with double-layer muscular walls, 630 × 120 with oval cephalic ganglion in middle part. Lemnisci thin, ribbon-shaped, convoluted, 712 × 38, longer than proboscis receptacle. Vagina with two sphincters. Posterior body end slightly retracted. Genital pore terminal.

**Figure 7.**
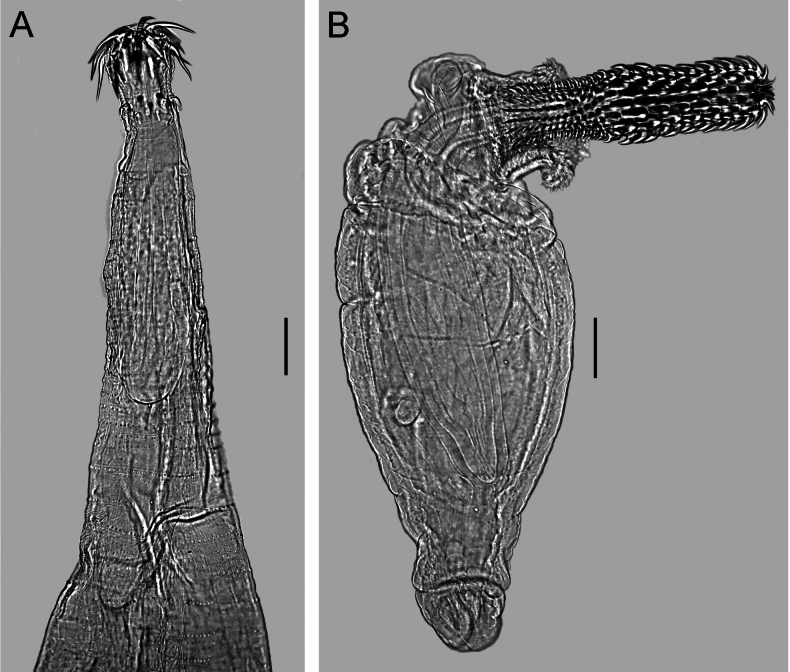
Light microscope photographs **A** anterior part of male of *Raosentis* sp. ex *Mystusmysticetus* Roberts from Thailand **B** total view on cysthacanth of *Arhythmorhynchus* sp. ex body cavity of *Mystusalbolineatus* Roberts from Thailand. Scale bars: 100 µm.

###### Remarks.

The definitive hosts of these acanthocephalans are gulls and waders (Charadriiformes), and fish are paratenic hosts. To date, 24 valid species have been described in the genus *Arhythmorhynchus* (Amin 2013). Only three species have more than 20 hooks in a longitudinal row on the proboscis: *A.xeni* Atrashkevich, 1978 described from Terek sandpiper *Xenuscinereus* (Güldenstädt, 1775) in Siberia, *A.longicollis* (Villot, 1875) Lühe, 1912 described from gulls in Europe, and *A.limosae* Edmonds, 1971 described from godwit *Limosalapponica* in Townsville, Queensland, Australia (Golvan 1956; Edmonds 1971; Atrashkevich 1978). The cystacanths of *Arhythmorhynchus* sp. from Thailand differs from all three species in the number of longitudinal rows of hooks (16 in *Arhythmorhynchus* sp. vs 19–20 in *A.xeni* and *A.limosae*, 22–24 in *A.longicollis*), as well as in the ratio of large spine-shaped hooks (8–9 and 12–13 in *Arhythmorhynchus* sp., 14–15 and 11–12 in *A.xeni* and *A.limosae*, respectively). The number of large hooks is similar in *Arhythmorhynchus* sp. and *A.longicollis*, 8–9 and 9–10 respectively; however, the length of the blades of the largest hooks in *Arhythmorhynchus* sp. is smaller than in *A.longicollis* (40–41 vs 48–50). Thus, morphological differences prevent us from classifying *Arhythmorhynchus* sp. as any of the known species of the genus.

### ﻿Molecular phylogeny

In total, 24 new sequences of four genetic markers for three species of Acanthocephala from freshwater fishes in Thailand were generated and deposited in GenBank (Acc. nos. PQ636375–PQ636382, PQ636383–PQ636389, PQ636390–PQ636392, PQ631040–PQ631045). Intraspecific genetic variability was observed only for the COI marker and was limited to a single substitution for both A. (A.) barbonymi sp. nov. and *P.rexus.* The BLASTn comparison of the nuclear markers showed the highest similarity of A. (A.) barbonymi sp. nov. with isolates of A. (A.) cf. tilapiae and A. (A.) bilaspurensis (96.5–97.5% for 18S rRNA gene and 90.0–90.3% for 28S rRNA gene), while COI sequences of the new species were 99.0% identical with A. (A.) kenyirensis (MN833316). The best matches of *P.rexus* sequences were 98.6% similarity in 18S rRNA gene with *P.nandai* (MW164853, MW164854), 94.8% similarity in 28S rRNA gene with *Pallisentis* sp. (MW421633), and 72.8% similarity with *P.celatus* (Van Cleave, 1928) (NC_022921).

**Figure 8.**
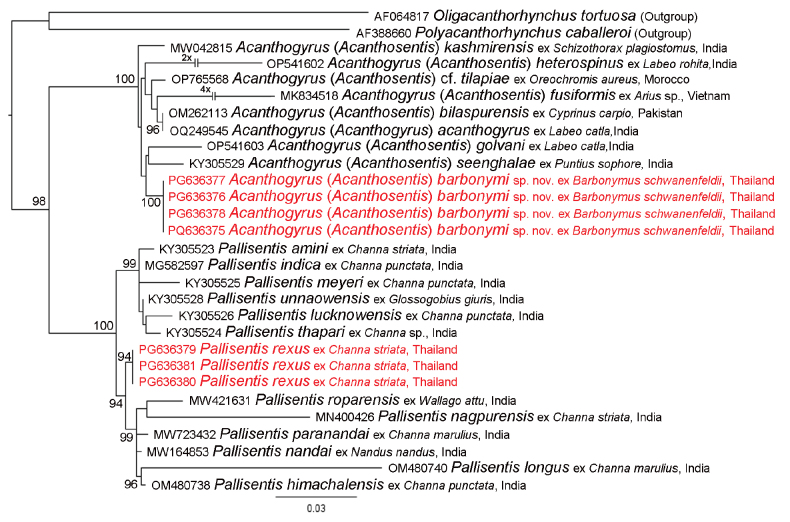
Phylogenetic relationships within the family Quadrigyridae inferred from the dataset of the 18 rRNA gene marker. The values near nodes were calculated by ultrafast bootstrapping in IQtree, only supports > 90 are shown. Scale bar indicates number of substitutions per site.

Phylogenetic analysis of the family Quadrigyridae clearly defined two highly supported clades. The first clade grouped isolates of *Acanthogyrus*, including A. (A.) barbonymi sp. nov.; however, weak support of internal nodes did not allow to define interrelationships within this genus. The second clade grouped the isolates of *Pallisentis*, and *P.rexus* formed well-supported sister lineage to the clade of *P.nandai* (MW164853), *P.paranandai* (MW723432), *P.roparensis* (MW421631), *P.nagpurensis* (MN400426), *P.himachalensis* (OM480738), and *P.longus* (OM480740) (Fig. 8).

## ﻿Discussion

The list of fish acanthocephalans in Thailand has been expanded to ten species with the addition of the two taxa characterized in this work, the new species A. (A.) barbonymi sp. nov., and a putative new species of *Raosentis*, the genus currently containing seven described species (Naidu 2012). *Pallisentisrexus* was originally described from *C.striata* in Chiang Mai basin in Thailand and later recorded in Taiwan. *Arhythmorhynchus* sp. previously had been reported from the goonch *Bagariusbagarius* (Hamilton) from the Mekong River in Chiang Rai province, Thailand (Purivirojkul and Areechon 2008), but the authors did not provide a description nor drawings, so we cannot compare our species with the acanthocephalans described in that study. In total, three species of *Pallisentis*, two species of the subgenus Acanthosentis, and five other genera are currently known from fishes in Thailand. This reflects a paucity of parasitological surveys of fish in this geographical area. A greater number of taxa has only been recorded in Vietnam, where 15 species have been found in freshwater teleosts (Arthur and Te 2006; Amin et al. 2014): *Acanthocephalusparallelcementglandatus* Amin, Heckmann & Ha, 2014, Acanthogyrus (Acanthosentis) indicus (Tripathi, 1959), *Cathayacanthusbagarii* Moravec & Sey, 1989, *Cleaveiuslongirostris* Moravec & Sey, 1989, *Dendronucleata* spp., *Micracanthorhynchinahemiculterus* (Demshin, 1965), Neoechinorhynchus (Hebesoma) spiramuscularis Amin, Heckmann & Ha, 2014, *Neotegorhynchus* (as *Brentisentis*) *cyprini* (Yin & Wu, 1984), *Pallisentis* spp., *Paradentitruncuslongireceptaculis* Moravec & Sey, 1989, *Pseudoacanthocephalusconiformis* Amin, Heckmann, Ha, 2014, and *Pseudorhadinorhynchusvietnamensis* Moravec & Sey, 1989. Two species of fish, *M.albolineatus* and *B.schwanenfeldii* have been added to the list of fish that are hosts of acanthocephalans. Considering that only 20 species of fish have been confirmed as hosts, species diversity of acanthocephalans in freshwater fishes in Thailand remains largely unexplored.

The new species A. (A.) barbonymi sp. nov. is described herein from *B.schwanenfeldii*, the same host from which three other members of the genus were described recently in Malaysia (Mohd-Agos et al. 2021). These authors provided morphological characterization of the three; however, available genetic data are inconsistent and hardly comparable with ours (see Remarks). As our requests to borrow type specimens have not been met to date, the specimens collected in Thailand were described as a new species based on significant morphological differences from the three species from Malaysia.

Amin et al. (2000) published a taxonomic revision of the genus *Pallisentis*, in which three subgenera *Pallisentis*, *Brevitritospinus*, and *Demidueterospinus* Amin, Heckmann, Ha, Luc & Doanh, 2000 were erected based mostly on the relative proportions of their proboscis hooks. However, their validity has not been supported by recent molecular phylogenetic analyses (Chaudhary et al. 2019; Gautam et al. 2020; Amin et al. 2021; Rana and Kaur 2021). Similarly, our phylogenetic analysis indicates that the subgenera *Brevitritospinus* and *Pallisentis* are not natural, monophyletic groups; two available isolates of the subgenus Brevitritospinus clustered with three isolates of *Pallisentis*.

Golvan (1959) listed *Acanthogyrus* Thapar, 1927 and *Acanthosentis* Verma & Datta, 1929 as subgenera of *Acanthogyrus.*Amin (1985) accepted their subgeneric statuses, while Golvan (1994) considered *Acanthogyrus* and *Acanthosentis* to be separate genera. Herein, we follow the most recent classification of Amin (2013), where these two taxa are listed as subgenera, which may be distinguished based on the number of proboscis hooks: six hooks in each of three rows in *Acanthosentis* and eight hooks in each of three rows in *Acanthogyrus*. In our phylogenetic analysis, the type species of the subgenus Acanthogyrus, A. (A.) acanthogyrus and eight species of the subgenus Acanthosentis, including A. (A.) barbonymi sp. nov., were clustered in a well-supported clade. However, low supports of internal nodes did not allow us to estimate phylogenetic interrelationships within the clade and prevent us to confirm or refute the validity of these subgenera.

It is obvious that further research is necessary to resolve phylogenetic relationships within the order Gyracanthocephala. The currently available genetic data are not sufficient to provide comprehensive phylogenetic hypothesis due to the low coverage of taxa and possible incongruence of genetic markers. Another obstacle is the general absence of vouchers in museum collections available for the evaluation of morphological structures. The generation of the sequences of multiple genetic markers that are linked to morphological vouchers deposited in international collections reachable by all taxonomists needs to be implemented (see Perrot-Minnot et al. 2023).

Finally, this work provides detailed morphological characterization and genetic data for several taxa, enriching our knowledge about the acanthocephalan fauna of fishes in Thailand. Moreover, both hosts, *C.striata* and *B.schwanenfeldii*, are commercially important fishes used as food and ornamental fish, respectively. The heavy infestation of these fishes with acanthocephalans observed in this study suggests that these parasites may have a negative impact on fish farming in Thailand.

## Supplementary Material

XML Treatment for Acanthogyrus (Acanthosentis) barbonymi

XML Treatment for Pallisentis rexus

XML Treatment for Raosentis

XML Treatment for Arhythmorhynchus

## ﻿Additional information

### Author ORCIDs

Olga Lisitsyna https://orcid.org/0000-0002-2975-3300

Mikuláš Oros https://orcid.org/0000-0002-0518-5708

Alexis Ribas https://orcid.org/0000-0002-1256-3316

Srisupaph Poonlaphdecha https://orcid.org/0000-0002-9434-3703

Daniel Barčák https://orcid.org/0000-0002-5975-0899

### Data availability

All of the data that support the findings of this study are available in the main text or Supplementary Information.

## Supplementary materials

Supplementary material 1 Voucher specimens and their deposition Data type xlsx https://binary.pensoft.net/file/1298971This dataset is made available under the Open Database License (http://opendatacommons.org/licenses/odbl/1.0/). The Open Database License (ODbL) is a license agreement intended to allow users to freely share, modify, and use this Dataset while maintaining this same freedom for others, provided that the original source and author(s) are credited.Daniel Barčák, Olga Lisitsyna
